# Identification of miRNA signatures associated with radiation-induced late lung injury in mice

**DOI:** 10.1371/journal.pone.0232411

**Published:** 2020-05-11

**Authors:** Claude J. Rogers, Agnes I. Lukaszewicz, Jason Yamada-Hanff, Ewa D. Micewicz, Josephine A. Ratikan, Mark A. Starbird, Thomas A. Miller, Christine Nguyen, Jason T. Lee, Tove Olafsen, Keisuke S. Iwamoto, William H. McBride, Dörthe Schaue, Naresh Menon

**Affiliations:** 1 ChromoLogic LLC, Monrovia, California, United States of America; 2 Department of Radiation Oncology, University of California Los Angeles, Los Angeles, California, United States of America; 3 Crump Institute for Molecular Imaging, University of California Los Angeles, Los Angeles, California, United States of America; ENEA Centro Ricerche Casaccia, ITALY

## Abstract

Acute radiation exposure of the thorax can lead to late serious, and even life-threatening, pulmonary and cardiac damage. Sporadic in nature, late complications tend to be difficult to predict, which prompted this investigation into identifying non-invasive, tissue-specific biomarkers for the early detection of late radiation injury. Levels of circulating microRNA (miRNA) were measured in C3H and C57Bl/6 mice after whole thorax irradiation at doses yielding approximately 70% mortality in 120 or 180 days, respectively (LD_70/120 or 180_). Within the first two weeks after exposure, weight gain slowed compared to sham treated mice along with a temporary drop in white blood cell counts. 52% of C3H (33 of 64) and 72% of C57Bl/6 (46 of 64) irradiated mice died due to late radiation injury. Lung and heart damage, as assessed by computed tomography (CT) and histology at 150 (C3H mice) and 180 (C57Bl/6 mice) days, correlated well with the appearance of a local, miRNA signature in the lung and heart tissue of irradiated animals, consistent with inherent differences in the C3H and C57Bl/6 strains in their propensity for developing radiation-induced pneumonitis or fibrosis, respectively. Radiation-induced changes in the circulating miRNA profile were most prominent within the first 30 days after exposure and included miRNA known to regulate inflammation and fibrosis. Importantly, early changes in plasma miRNA expression predicted survival with reasonable accuracy (88–92%). The miRNA signature that predicted survival in C3H mice, including miR-34a-5p, -100-5p, and -150-5p, were associated with pro-inflammatory NF-κB-mediated signaling pathways, whereas the signature identified in C57Bl/6 mice (miR-34b-3p, -96-5p, and -802-5p) was associated with TGF-β/SMAD signaling. This study supports the hypothesis that plasma miRNA profiles could be used to identify individuals at high risk of organ-specific late radiation damage, with applications for radiation oncology clinical practice or in the context of a radiological incident.

## Introduction

Radiation injury can affect any organ, with responses being dose- and time-dependent. Classic tissue-specific acute radiation syndromes (ARS) after moderate doses of total body irradiation (TBI) develop as the hematopoietic or gastrointestinal system fails within weeks or days following exposure, respectively. If mortality is avoided during the acute phase, delayed effects of acute radiation exposure (DEARE) can manifest within months or years after exposure, with lung and heart damage being classic examples of sites for chronic radiation pathologies. Biomarkers to predict both acute and late adverse outcomes of radiation exposure are critically needed to identify individuals at risk after a radiological incident, especially since early therapeutic intervention can dramatically improve survival rates in the context of ARS [1–6], and perhaps late disease [2]. Efforts into monitoring changes in blood or plasma have yielded encouraging data and identified potential biomarkers of radiation exposure, including proteins [7–10], mRNA [11–16], non-coding RNA [17], and microRNA (miRNA) [18–24]. Because circulating miRNA are protected from degradation in extracellular vesicles or protein complexes, and are stable at room temperature for hours to days, they are exceptionally well suited for triage in the field [25].

miRNA are small (19–22 nt), non-coding nucleotides that post-transcriptionally regulate protein expression [26]. Approximately 1–2% of all genes in mammals encode for miRNA. miRNA can be released into extracellular biofluids, such as plasma, and may participate in cell-cell and tissue-tissue communication [27]. Changes in the circulating miRNA profile have been observed in pathophysiological conditions, such as cancer [28,29], and after exposure to radiation [18–24]. Since miRNA can be tissue-specific, the circulating miRNA profile may reflect what tissues are injured. While this field is still in its infancy, TBI drives significant alterations in circulating hematopoietic miRNA biomarkers, such as miR-150-5p, and the extent of dysregulation correlates well with the later onset of neutropenia and lymphopenia in an individual animal [19–21].

Finding tissue damage-specific miRNA signatures may be particularly important for assessing the likelihood of DEARE in a radiological event as varying degrees of partial body shielding is likely which would result in non-uniform doses to different organs in the body. This may increase survival from ARS but simultaneously increase the incidence of late radiation injury. Well-defined partial body exposures of animals can be used to model such circumstances and investigate tissue-specific consequences. Here, we use whole thorax lung irradiation (WTLI) for late radiation injury to the heart and lung with shielding to prevent ARS mortality. Since such responses are in part genetically defined and vary with the mouse strain [30–32], we used C3H mice that develop peak radiation pneumonitis approximately 3–4 months after exposure without progression into fibrosis [33] and C57Bl/6 mice that present with *bona fide* pulmonary fibrosis 6 months after exposure [30]. As these are different endpoints, longitudinal temporal changes in circulating miRNA were assessed after doses of WTLI that were estimated to cause death in ~70% of the mice at times associated with these endpoints. Local and systemic radiation damage was also monitored by changes in body weight, hematology, microcomputed tomography (microCT), and histology. We observed that radiation caused strain-specific major changes in systemic circulating miRNA profiles and persistent alterations in local miRNA profiles in irradiated lungs. Our results indicated that miRNA profiling may be used to predict late organ injury long before the manifestation of symptoms.

## Materials and methods

### Whole Thorax Lung Irradiation (WTLI)

All animal procedures were performed at the University of California, Los Angeles, under institutional IACUC approval (ARC # 1999–173) in accordance with the Animal Welfare Act and the Guide for the Care and Use of Laboratory Animals of the National Institutes of Health [34]. Animals were observed daily throughout the course of the experiment and defined criteria for premature euthanasia (loss of body weight >20%, body conditioning score of 2, labored breathing, and decreased mobility) were closely followed. Gnotobiotic C3Hf/Sed/Kam (C3H, N = 96) and C57Bl/6 (N = 96) mice 8 to 12 weeks old were exposed to a single dose of local radiation to the thorax estimated to be lethal for 70% of mice within 120 days for C3H (LD_70/120_; 13.92 Gy), and within 180 days for C57Bl/6 mice (LD_70/180_; 13.99 Gy). These estimates come from institutional lethality probit curves derived under identical conditions to those used in the experiments. The two strains differ in the endpoint they express and therefore the dose that is required to reach that endpoint. Equal numbers of female and male mice were used to study gender differences. Prior to irradiation, mice (8–10 week-old males, 10–12 week-old females) were randomized into groups for an average body weight of 29 ± 1 g. Mice were anesthetized using an intraperitoneal injection a mixture of ketamine and xylazine (80 mg/kg ketamine and 4 mg/kg xylazine) and an ophthalmic ointment was applied to prevent corneal drying (Puralube^®^ vet ointment, Dechra Veterinary Products, Northwich, UK). Anesthetized mice were placed in a jig (S1 Fig) with the thorax positioned so that a 2.75 cm wide strip was exposed to X-rays administered vertically with a focus-to-surface distance of 32 cm with the rest of the body shielded with 3 mm lead that gives 99.5% attenuation. The jig was centrally located within a field of 26.4 cm diameter. Using a Gulmay Medical RS320 Irradiation System X-ray unit operated at 300 kV (Gulmay Medical Ltd.; Camberly, Surrey, UK) with a permanent 4 mm Be filter, and the beam was filtered using 1.5 mm Cu and 3 mm Al giving a HVL of 3mm Cu, and a dose rate of 1.568 Gy/min. Dosimetry was performed by certified medical physicists using a 0.6 cc Farmer Ionization Chamber (PTW N30006), Harshaw TLD-100H (LiF:Mg, Cu, P) and self-developing film (GAFCHROMIC EBT2, International Specialty Products, Wayne, NJ) calibrated against a clinical ^60^Co irradiator (Theratron-1000, MDS Nordion, Ontario) for NIST standardization, and a phantom mouse with TLD dosimeters. Field homogeneity was verified to be within 1%. Additional self-developing film was placed under batches of animals to confirm accurate radiation dosimetry on the day of experiment.

### Blood collection and hematology

Blood was sampled 14 days prior to irradiation, 2 or 5 days after exposure, at monthly intervals thereafter, and at the end of the experiment. For live mice, retro-orbital bleeding into EDTA-coated glass capillaries after isoflurane anesthetization was used and the volume of blood collected did not exceed 1.25% of the body weight, yielding on average 100 μL. A 25 μL aliquot of blood was analyzed for complete blood count (CBC) using the Hemavet system (Drew Scientific, Inc.). Plasma was separated from leukocytes (WBC) and erythrocytes (RBC) following a 20 minute centrifugation at 400 g and stored in aliquots at −80°C until use. Terminal bleeds taken from euthanized mice from the heart were treated similarly.

### microCT and histopathology

Loss in aerated lung volume can be analyzed by microCT and correlates well with radiation-induced lung damage [35,36]. Animals reaching the study endpoint (C3H: day 150, C57: day 180) were subjected to CT scanning (MicroCAT II CT Scanner, Siemens Preclinical Solutions, Knoxville, TN) under continuous isoflurane (1–2% in O_2_) anesthesia. Images were acquired with the X-ray source biased at 80 kVp and 500 μA with 360 projections, exposure time of 500 ms, detector bin mode of 4 × 4, and an effective pixel size of 0.2 mm. The total scan time was about 1 min with 5–10 min image reconstruction. Images were analyzed with the open-access Medical Image Data Examiner AMIDE (http://www.gnu.org/copyleft/gpl.html) to select the region of interest (ROI) and to quantify the volume of aerated lung volume excluding the esophagus (3D-isocontour threshold < −400 HU). After completion of CT scanning, animals were euthanized, cardiac blood collected, and the lung perfused with PBS followed by formalin and sent for processing by the Translational Pathology Core at UCLA. Paraffin-embedded tissues were sectioned and stained with hematoxylin and eosin and/or Masson’s trichrome for histopathological assessment by an expert pathologist. Selected mice had tissues taken after PBS infusion and snap frozen for miRNA analysis (see below).

### miRNA analysis by next-generation sequencing

Plasma was checked for excess hemolysis using UV-vis spectroscopy and samples with an absorbance value greater than 1.2 A.U. at 415 nm, corresponding to 0.3% hemolysis [37], were excluded from the study. Circulating miRNA was isolated from 25 μL of plasma using the miRCIRY RNA Isolation Kit (Biofluids; Exiqon). Tissue miRNA was extracted from 20 mg of homogenized tissues in QIAzol (Qiagen) with the miRNEasy Kit (Qiagen). Libraries for sequencing were prepared using the QIAseq miRNA Library Kit; (Qiagen) with 5.8 μL of miRNA extracts as input, a 1:5 dilution of the 3’-adaptor, a 1:2.5 dilution of the 5’-adaptor, a 1:5 dilution of the RT primer, and 22 amplification cycles. The concentrations of the prepared libraries were determined via Bioanalyzer analysis (2100 Electrophoresis Bioanalyzer, Agilent). Libraries with an adapter dimer peak (~160 nt) at least five times greater than the library peak (~180 nt) were not sequenced. miRNA counts for 2 nM samples were determined via next-generation sequencing (NextSeq 550, Illumina) using 76 read cycles. Demultiplexing, trimming (read lengths between 18–40 bp, 5’-end base quality ≥ 30, read score ≥ 20, and 3’-end adaptor sequence to trim of AACTGTAGGCACCATCAAT), and miRNA alignment (using “Mus musculus/mm10” as the species) was performed using BaseSpace (Illumina), using the applications Small RNA v1.0.1, FASTQ Toolkit v2.2.0, and FASTQ Generation v1.0.0. Sequencing runs with less than 100,000 miRNA reads were rejected.

### Data analysis

Raw sequencing counts were normalized by total library size to obtain the reads for a given sequence per million total reads (RPM), then by quantile normalization of the log_2_ RPM. Differential expression analysis was performed in R (version 3.4.3) using normalized counts using the limma and voom software packages (version 3.28.10) [38]. KEGG pathways associated with differentially expressed miRNA were identified using mirPath v.3 software [39]. miRNA that correlated with survival were identified using Cox’s proportional hazard method or linear regression using an elastic net penalty. Kaplan-Meier survival functions were compared using the log rank test (Mantel-Cox) [40], and uncertainties (95% confidence intervals) estimated according to Greenwood [41]. Blood count data were compared over time using an ANOVA model, and p-values were obtained by comparing post-irradiation values to pre-irradiation values using Dunnett’s method.

### Animal study ethical statement

University of California, Los Angeles’s IACUC-approved protocols and NIH guidelines and defined criteria for premature euthanasia were followed. The experiments were approved under the IACUC protocol #1999–173.

## Results

### Hematological changes after WTLI

**Fig 1:**
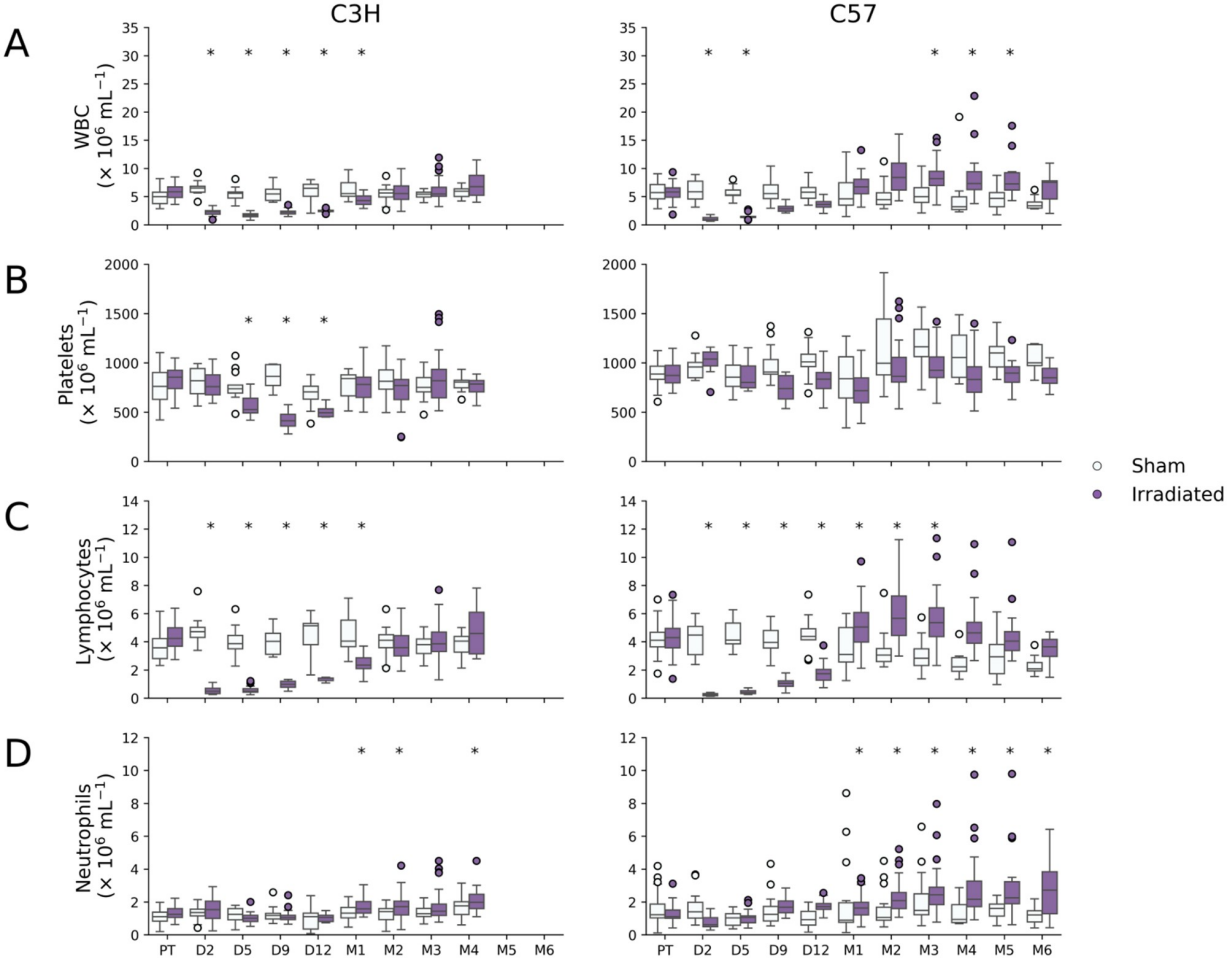
Blood cell counts for WTLI and sham irradiated C3H and C57 mice. WTLI resulted in a rapid drop in (A) white blood cells (WBC) and (C) lymphocytes in both strains of mice that recovered by month 2 in C3H mice and became slightly elevated by month 1 in C57 mice. (B) Platelets were slightly reduced in C3H mice between days 5 and 12. (D) Neutrophil counts were elevated in both strains by month 1, but more persistently in C57Bl/6 mice. Statistically significant changes in counts compared to pre-radiation levels (Dunnett’s test p-value < 0.001) are indicated by an asterisk.

Irradiated C3H and C57Bl/6 mice experienced the characteristic decline in total WBC (3–5-fold; p-value < 0.0001) and lymphocyte (8–10-fold, p-value < 0.001) counts within the first few days after WTLI (Fig 1A and 1C). Decrease in blood cells after partial body irradiation is generally due to radiation killing as the blood recirculates rapidly through the irradiated field. Lymphocyte and total WBC counts returned to baseline 1–2 months after WTLI, with C57Bl/6 mice recovering earlier than C3H mice and overshooting basal levels. Lymphocytes remained elevated in some C57Bl/6 mice for up to 5 months (1.4-fold, p-value < 0.001). Neutrophil numbers also showed a similar compensatory increase starting one month post-irradiation and remained elevated by as much as 1.6–2.5-fold in both strains (p-values < 0.001; Fig 1D). Platelets were less affected in C57Bl/6 mice than C3H with declines and increases less marked than for other blood cells (Fig 1B).

### Changes in survival and body weight after WTLI

**Fig 2:**
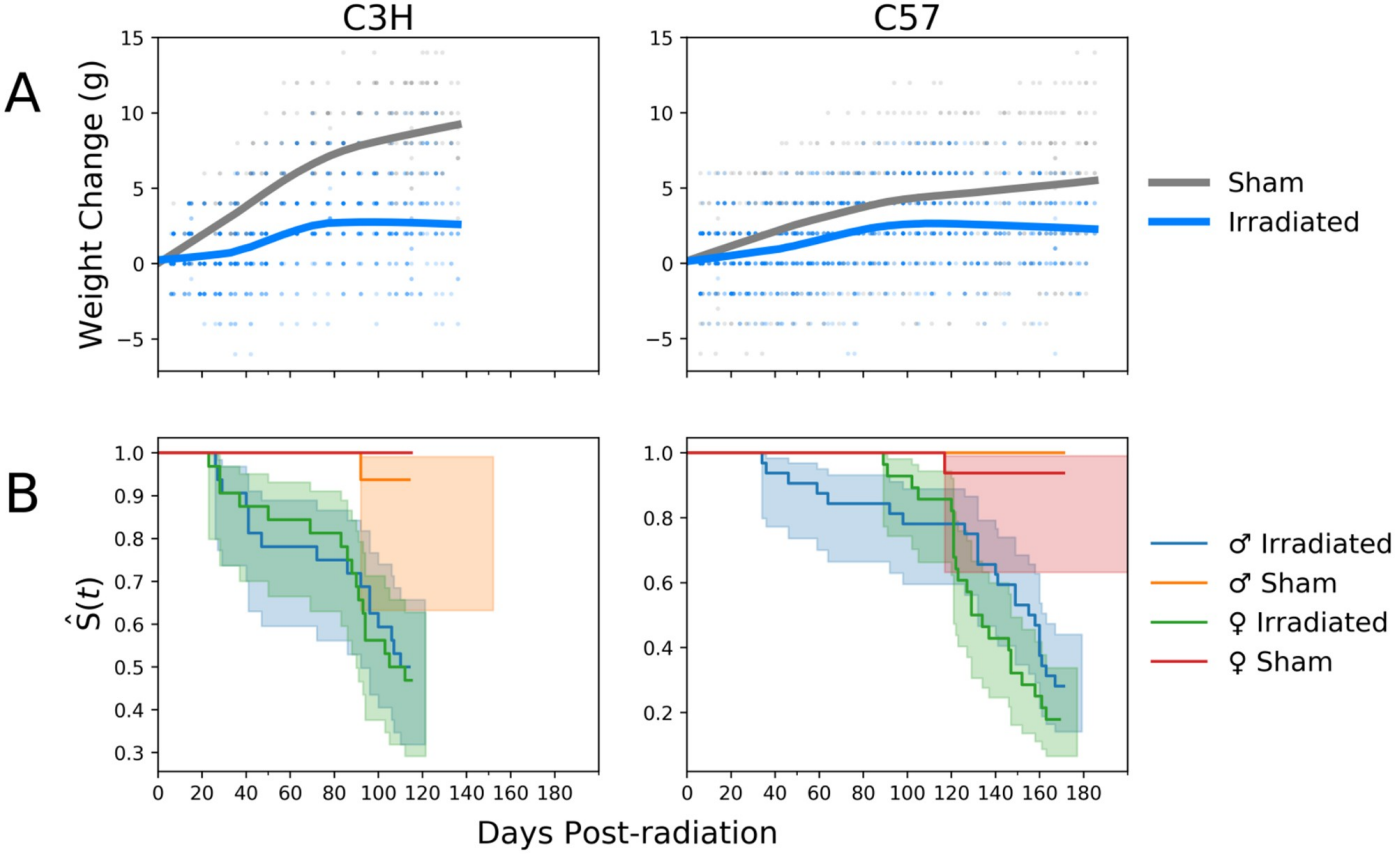
Changes in weight and Kaplan-Meier survival curves for WTLI and sham irradiated C3H and C57 mice by gender. (A) Irradiated mice failed to gain weight compared to sham irradiated controls in both strains. (B) Kaplan-Meier curves show no gender-based differences (C57: p-value = 0.1, C3H: p-value = 0.8, log rank test), with C57 mice surviving longer than C3H, on average.

Other phenotypic indications of radiation-induced injury included a failure to gain weight at the rate of sham irradiated controls, especially over the first 40 days after exposure and again after day 80 (two-way ANOVA p-values < 0.001, Fig 2A). Overall survival for irradiated animals was consistent with the institutional lethality profile, with 48% of C3H mice surviving until day 120 and 23% of the C57Bl/6 strain surviving until day 180 (Fig 2B) without statistically significant gender differences (C57Bl/6: p-value = 0.1, C3H: p-value = 0.8, log rank test).

### Characterization of thoracic injury using histology and microCT

**Fig 3:**
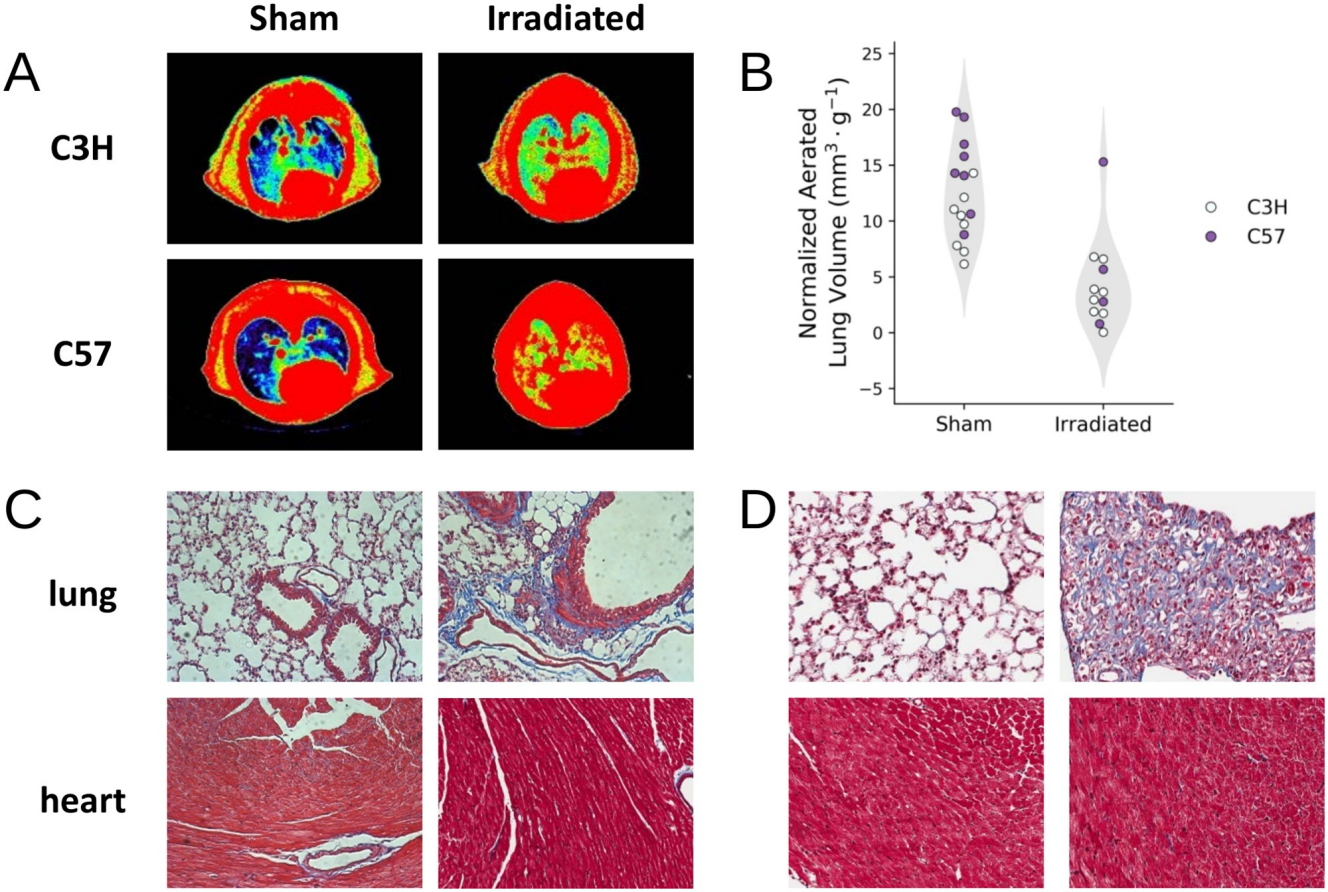
Radiation-induced lung injury was observed in C3H and C57 mice. (A) CT images of the mouse thorax pseudo-colored based on radio-opacity, with the color grading from blue to red representing increased opacity. Using radio-opacity to approximate tissue density, aerated lung volume (blue) is reduced in irradiated animals compared to controls. (B) Quantification of the aerated lung volume, normalized by the animal weight, show that there was a statistically significant reduction in aerated lung volume in irradiated animals (p-value < 0.05). (C and D) Histological sections of C3H (C) and C57Bl/6 (D) heart and lung tissue stained with Masson’s trichrome.

Cohorts of mice reaching the study endpoint (N = 16 C3H; N = 12 C57Bl/6) were examined for radiation-induced heart and lung damage as assessed by microCT and histology. Significant loss of aerated lung volume was widespread amongst irradiated C3H (female) and C57 (male) mice, consistent with the development of radiation-induced pneumonitis or fibrosis (Fig 3). On average, aerated lung volumes relative to total body weight decreased 2.8- and 2.4-fold in C3H and C57 mice, respectively, compared to sham irradiated control animals (p = 0.00017 and p = 0.013). These findings were supported by histopathology detecting obvious signs of cardiac and pulmonary damage in both strains of mice. In lungs, both strains had some elevated collagen deposits as visualized by Masson’s trichrome stain, but this was more consolidated in the C57Bl/6 strain, while the C3H strain showed pneumonitis (Fig 3).

### Differentially expressed miRNA in tissues

**Fig 4:**
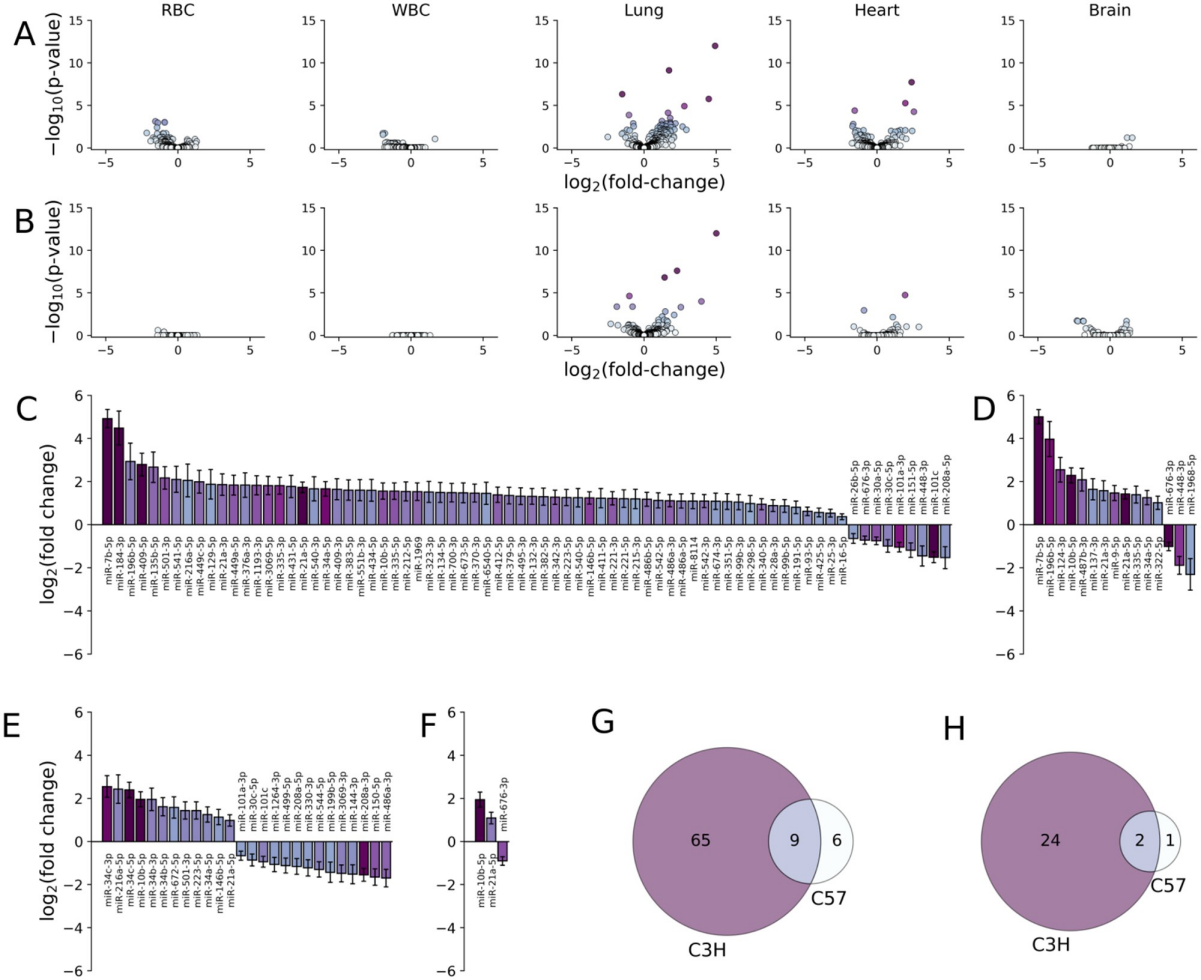
Differentially expressed miRNA in tissues from irradiated mice at the study endpoint compared to controls. Volcano plots showing the log_2_ fold-change versus–log_10_ p-value for each miRNA in C3H (n = 15, at month 4; A) and C57 (n = 20, at month 6; B) mice extracted from the indicated tissue. Significant changes in miRNA expression was observed in lung tissue 4 (C3H) and 6 (C57) months after WTLI. (C and E) Log_2_ fold change in expression for miRNA in lung tissue with statistically significant changes in expression (p-value < 0.05) in WTLI versus control C3H and C57 mice, respectively. (D and F) Log_2_ fold change in expression for miRNA in heart tissue with statistically significant changes in expression (p-value < 0.05) in WTLI versus control C3H and C57 mice, respectively. (G and H) Venn diagrams for miRNA expressed in lung and heart tissue (respectively) in C3H and C57 mice.

Differential expression analysis of next-generation miRNA sequencing data extracted from whole lung and heart homogenates at 4 (C3H) or 6 (C57Bl/6) months post-WTLI revealed significant alterations in miRNA abundance compared to sham irradiated controls, respectively. More changes in miRNA abundance were observed in irradiated C3H mice in both the lung (74 sequences) and heart (26 sequences) than in irradiated C57Bl/6 mice (15 and 3 sequences, respectively, at a p-value cutoff of 0.05 and an absolute fold-change of at least 2.25-fold; Fig 4A–4F). Overlapping tissue-specific radiation-induced patterns of differentially expressed miRNAs that were common to both strains included miR-7b-5p, -10b-5p, -21a, and -34a-5p in lungs and miR-10b-5p and -21-5p in hearts (Fig 4G and 4H). Other tissue-specific responses were similar between these two strains, with C3H mice presenting alterations in RBCs and WBCs miRNA profiles (11 and 3 sequences, p < 0.05) and C57Bl/6 presenting fewer (Fig 4A and 4B). Brain tissue miRNA profiles in C3H and C57Bl/6 showed a similar, relatively minor, response to radiation at these time points (Fig 4A and 4B).

### Differentially expressed circulating miRNA

**Fig 5:**
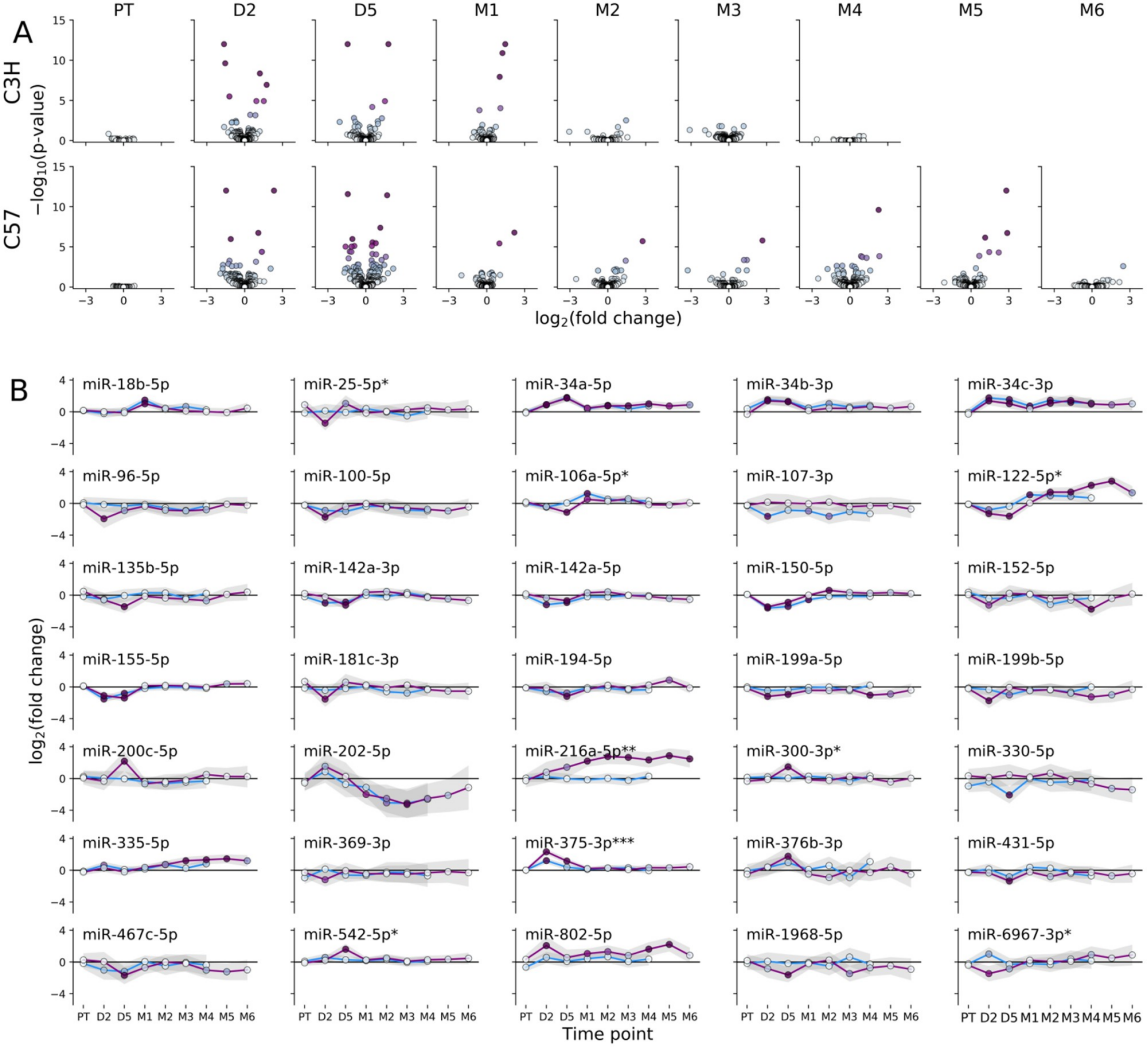
Differentially expressed miRNA in plasma from irradiated mice during the first four to six months after exposure. (A) Volcano plots showing the log_2_ fold-change versus–log_10_ p-value for plasma miRNA in WTLI versus sham irradiated controls at the indicated time points and strains. (B) Temporal expression profile for differentially expressed miRNA (> 2.5-fold change in expression and p-value < 0.05) in plasma for C3H (blue line) or C57 (purple line) mice. Statistical differences between strains were evaluated by two-way ANOVA and indicated with an asterisk (* indicates 0.005 ≤ p-value < 0.05, ** indicates 0.0005 ≤ p-value < 0.005, *** indicates p-value < 0.0005).

Circulating plasma miRNA profiles were studied over time after WTLI for comparison with the tissue profiles. In C3H mice, differential expression analysis indicated significant changes in the circulating miRNA profile within 48 h of exposure that persisted until day 30, returning to levels consistent with unirradiated controls by day 60 (Fig 5A). C57Bl/6 mice showed a similar early response to irradiation (2 and 5 days) but by day 30, fewer miRNAs remained differentially expressed (e.g. miR-202-5p and miR-216a-5p). Interestingly, many of the differentially expressed miRNA showed similar temporal expression profiles in both strains (Fig 5B). For example, expression profiles of circulating miR-18b-5p, the miR-34 family members, miR-142a-3p and -5p, miR-150-5p, miR-155-5p, and miR-202-5p were nearly identical in pattern and magnitude for C3H and C57Bl/6 mice. Other miRNA, including miR-106a-5p, miR-122-5p, and miR-375-3p showed similar overall longitudinal patterns, albeit at different magnitudes (two-way ANOVA). The temporal abundance of a few miRNA sequences showed statistically significant strain differences, such as the longitudinal increase in miR-216a-5p abundance in C57Bl/6 mice alone. Of note, in this strain there was a second, delayed and persistent (day 90–150) increase in differentially expressed miRNA until the study endpoint for this strain (day 180), which was not observed in C3H mice within their specific endpoint (day 120; Fig 5A). Some differentially expressed circulating miRNAs were also differentially expressed in lung tissues: miR-335-5p and -34a-5p were differentially expressed in both strains; miR-135b-5p, -216a-5p, -431-5p, and -542-5p in C3H; and, miR-1968-5p was also downregulated in C57Bl/6. Similarly, the circulating markers miR-34a-5p, -34b-3p, -34c-3p, -150-5p, -199b-5p, and -216a-5p were also differentially expressed in C3H heart tissue.

### Functional roles of differentially expressed miRNA

**Fig 6:**
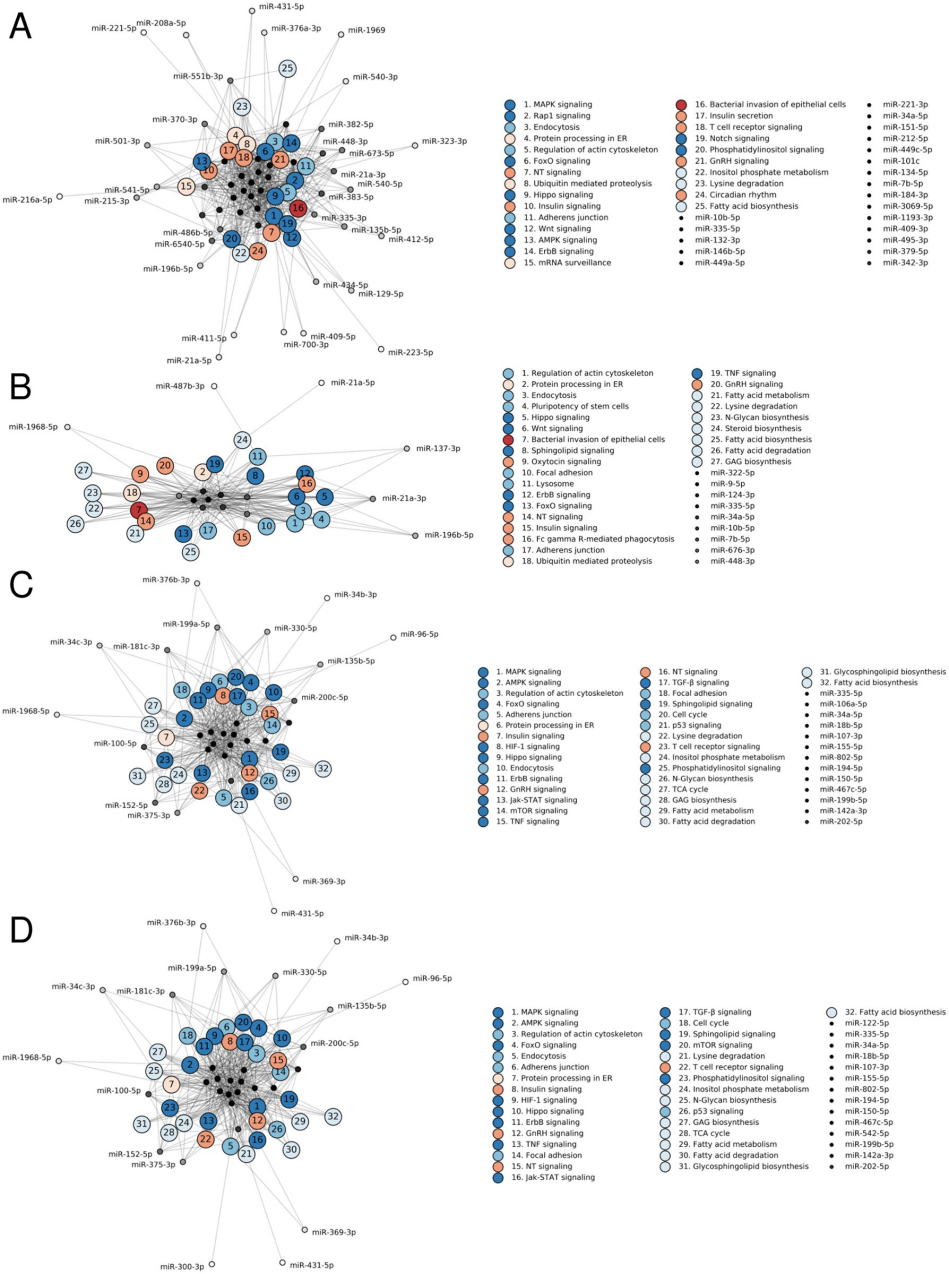
Predicted KEGG pathways for differentially expressed miRNA in lung tissue and plasma. KEGG pathways associated with differentially expressed miRNA in whole lung tissue from C3H (A) and C57 (B) mice and in plasma from C3H (C) and C57 (D) mice. KEGG pathways are sorted, from high to low, by the number of miRNA sequences predicted to interact with key pathway genes. Pathway nodes are colored by function, from blue to red: environmental information processing (i.e. signal transduction), cellular processes, metabolism, genetic information processing, organismal systems (i.e. immune and endocrine systems), and disease.

To dissect the mechanistic role of these miRNA, we examined the KEGG pathways associated with differentially expressed miRNA. This approach has been recently used to understand the role of differentially expressed miRNA following radiation injury in the rat esophagus and mouse lung [42,43]. While only about 20% of differentially expressed miRNA in plasma were also differentially expressed in irradiated lung tissue, the pathways in question are incredibly similar. The majority of the affected pathways are associated with signal transduction (e.g. FoxO, Hippo, and ErbB signal transduction, Fig 6). Immune system pathways (e.g. T cell receptor signaling) and the endocrine system (e.g. insulin and GnRH signaling pathways) were also implicated, as was metabolism (e.g. fatty acid biosynthesis, lysine degradation), and cellular processes (e.g. adherens junction, regulation of actin cytoskeleton; Fig 6). Similar pathways were predicted to be disrupted in the heart in both strains (e.g. MAPK signaling, regulation of actin cytoskeleton, and fatty acid metabolism). Indeed, all of the differentially expressed miRNA in the C57Bl/6 heart were also differentially expressed in the lung (Fig 4D and 4F).

### Predicting survival based on the circulating miRNA profile

**Fig 7:**
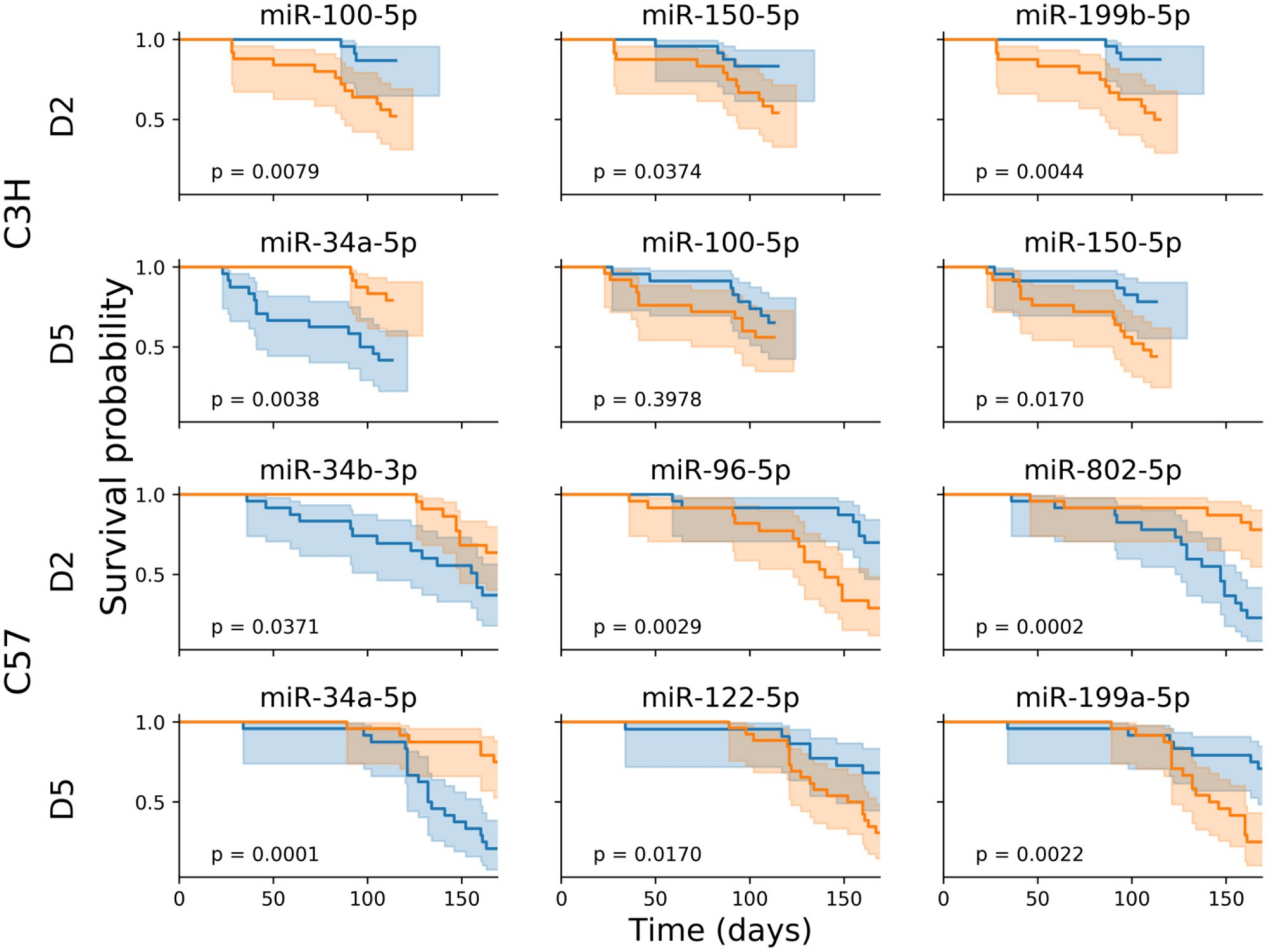
Differences in survival probability for mice with miRNA expression above or below the median abundance. For each strain and time point, key miRNA in a Cox proportional hazard model were identified. Mice were divided into two cohorts based on expression above (blue) or below (orange) the median abundance. In most cases, the difference in survival between these two cohorts was statistically significant (log rank test), suggesting a role for these miRNAs as potential biomarkers for early identification of late organ injury following exposure to ionizing radiation.

To identify miRNA that could predict the risk of an individual animal for developing severe late radiation tissue damage, we developed a Cox proportional hazards model for predicting survival on the basis of relative circulating miRNA abundance. For each strain, a model was fit to the miRNA expression data of differentially expressed miRNA at day 2, day 5, month 1, and month 2, and the ability of the model to rank the animals by hazard based on the miRNA profile at that time point was evaluated. Models that were able to predict survival with reasonable accuracy were identified using day 2 miRNA expression in C3H mice (92% accuracy) and day 5 miRNA expression in C57Bl/6 mice (89% accuracy). This is consistent with the peak in differential expression abundance for each strain (Fig 5A). The accuracy of models at the month 1 and 2 time points were slightly lower (83–87%; Table 1). With the models in hand, we examined the miRNA that contribute most strongly to the survival prediction, including miR-34a-5p, -34b-3p, -96-5p, -100-5p, -122-5p, -150-5p, -199a-5p, -199b-5p, and -802-5p. For each strain, mice were divided into two cohorts based on expression of these miRNA above or below the median abundance at the day 2 and 5 time points (Fig 7). In both C3H and C57Bl/6 mice, elevated expression of miR-34a-5p at day 5 was associated with reduced survival. Similarly, high levels of circulating miR-34b-3p at day 2 were associated with reduced survival in C57Bl/6 mice. Members of the miR-199 family, miR-199a-5p and -199b-5p, had lower circulating abundance in animals with reduces survival in C57Bl/6 mice at day 5 and C3H mice at day 2, respectively. Lower levels of miR-150-5p were associated with reduced survival in C3H mice at day 2 and 5. In these mice, miR-100-5p was important for predicting survival for both day 2 and 5, but only at day 2 was the difference statistically significant (log rank test) using median abundance as the threshold. In C57Bl/6 mice, high levels of miR-802-5p and low levels of miR-122-5p were associated with reduced survival at day 2 and 5, respectively.

**Table 1:** Accuracy of Cox proportional hazard models fit to miRNA expression profiles at the indicated time points. Optimism correction was performed using Harrell’s bootstrap method to minimize overfitting [44]. The accuracy is the ability of the model to rank mice in order of survival.

	PT	DAY 2	DAY 5	MONTH 1	MONTH 2
**C3H**	80%	92%	82%	87%	86%
**C57BL/6**	80%	88%	88%	85%	86%

## Discussion

Predicting late adverse reactions to radiation exposure long before they manifest opens the possibility for early intervention and could greatly improve clinical outcomes to accidental or therapeutic exposures to ionizing radiation. By using the well-characterized WTLI model in C3H and C57Bl/6 mice, we sought to link early changes in circulating miRNA abundance to the late onset of radiation-induced organ damage, especially late lung injury. Previous work with this model suggests that both C3H mice and C57Bl/6 mice have the tendency to develop pneumonitis while C57Bl/6 mice progress towards pulmonary fibrosis, which was consistent with CT imaging and histology from this study. Characteristic radiation hematologic toxicity exemplified in blood count nadirs and the later compensatory increases in lymphocyte and neutrophil counts seen in our irradiated animals are consistent with the literature on animal models and in humans, as are the other signs of radiation injury that were noted including inadequate weight gain and increased mortality [45]. Interestingly, the overshoot in circulating neutrophils and lymphocytes late after exposure is greater in C57Bl/6 mice than C3H mice and temporally correspond to the onset of radiation-induced pulmonary disease [30]. Taken together, our pathophysiological observations are consistent with previous studies in the literature.

Comparing miRNA abundance in radiation-damaged tissue, collected at the study endpoint for each strain, revealed a number of differentially expressed miRNA in lung tissue consistent with long-term dysregulation of relevant cellular processes in the affected tissue. In both strains of mice, miR-7b-5p, -196b-5p, -10b-5p, -21a, -335-5p, and -34a-5p were upregulated in irradiated animals and miR-448-3p and -676-3p were downregulated. Pathway analysis of these miRNA suggests roles in regulation of actin cytoskeleton, bacterial invasion of epithelial cells, and protein processing in the endoplasmic reticulum. The former two pathways lead to changes in actin polymerization and focal adhesion, consistent with the cellular response to stress and an open pathway towards fibrosis. Indeed, miR-196b-5p, -21a-3p, and -335-5p may regulate ROCK1/2 signaling, which is critical for many important cellular functions, including proliferation, migration, adhesion, and apoptosis/survival [46]. Highly relevant to this study is the observation that ROCK seems to be regulating actin polymerization in an experimental mouse model of lung fibrosis and in human subjects with idiopathic pulmonary fibrosis [47]. The fact that miR-335-5p was also overexpressed in plasma in our irradiated cohorts as early as two days post-irradiation supports the hypothesis that circulating miRNA profiles may contain specific signatures from radiation-damaged tissue. In addition to *Rock1*, miR-335-5p may interact with *Pik3r*, *Mapk*, *Camk2*, *Ep300*, and *Smad7*, and other genes known to be involved in fibrosis. Equally relevant in the context of radiation lung injury are differentially expressed miRNAs that are known to interact with genes regulating fatty acid and glycosphingolipid biosynthesis and by extension correlate with perturbations of surfactant lipid homeostasis, a tell-tale sign during the development of pneumonitis [48]. In fact, increases in levels of extracellular lipids was associated with decreases in *Fasn* and *Hmgcr* transcript levels [49], which are both predicted to interact with miR-335-5p. Consistent with this, differentially expressed miRNA are predicted to affect AMPK signaling which is one of the central regulators of cellular metabolism [50], in particular lipid biosynthesis. Overall, it appears that the changes in the miRNA profile in lung tissue seen here are consistent with known radiation-induced cellular and molecular fibrotic responses. Observations such as these provide the basis for further mechanistic studies into the role of miRNAs in radiation inflammation, fibrosis, and healing.

Approximately half of the miRNAs differentially expressed in the C3H heart were also affected in C3H lung, whereas this overlap between the miRNA signatures in heart and lung was 100% in C57Bl/6. It is not surprising, therefore, that similar pathways were predicted to be altered in both tissues. However, the radiation-induced differential expression appeared to be overall milder in the heart than in lung both in terms of the number of differentially expressed sequences and the magnitude of the fold change, which was true for either strain (Fig 4). This is consistent with our histology findings that suggest lung damage is more prevalent and severe than heart damage in both strains. The fact that the total number of differentially expressed miRNA in both tissues was higher in C3H mice than in C57 may be due to the difference in time point the tissue was examined (month 4 for C3H and month 6 for C57), since C3H mice tend to succumb from pneumonitis before the development of fatal fibrosis.

Similarly, early changes in circulating plasma miRNA were associated with genes that regulate cellular processes involved in lung damage and that can lead to pneumonitis or fibrosis. Several markers identified in this study have been observed to change after TBI in non-human primates, including up-regulation of miR-375 and -34a-5p, and down-regulation of miR-122-5p, -150-5p, and -100-5p [20,24]. This suggests that there may be an evolutionarily conserved molecular response to radiation injury that manifests itself as a characteristic systemic miRNA signature. Interestingly, similar patterns of dysregulation of these miRNAs occurred after different types of radiation injury (TBI versus WTLI) that typically result in death at drastically different time scales (< 60 days versus > 150 days, on average) and through different mechanisms. Notably, miR-34a-5p, -100-5p, and -150-5p were identified in survival analysis of C3H mice, but not C57Bl/6 (Fig 7). Pathway analysis of these miRNAs indicates roles in acute NF-κB-driven pro-inflammatory pathways. While these miRNAs were also differentially expressed in C57Bl/6 mice, these sequences were not identified in survival analysis for that strain. Instead, C57Bl/6 survival miRNA, including miR-122-5p and -802-5p, are implicated in TGF-β-SMAD signaling (i.e. fibrosis). Considering the differences in the cause of mortality between the two strains, the identification of miRNA associated with pro-inflammatory responses in C3H mice versus pro-fibrotic responses in C57Bl/6 mice by survival analysis, despite the similarity in the differentially expressed circulating miRNA between the strains (Fig 5B), underscores the potential utility of this class of molecules as predictive biomarkers.

Supporting this, Cox survival analysis of C57Bl/6 mice identified specific miRNA predicted to regulate the FoxO genes *Foxo1* and *Foxo3* (miR-802-5p and -96-5p) that are also implicated in regulating fibrogenesis [51]. Pathways associated with fibrogenesis may be affected by perturbations in miRNA expression in both strains even if only C57 present with *bona fide* fibrosis while C3H succumb to radiation pneumonitis before fibrosis can fully develop. For example, miRNAs miR-150-5p and -96-5p are predicted to regulate transcription factors associated with fibrosis, including *Hoxa9* and *Zeb1* (epithelial mesenchymal transition [52,53]); Collagen genes are predicted to associate with miR-150-5p (*Col1a1*), and miR-802-5p (*Col4a5*); and, miR-100-5p, -199a-5p, -199b-5p, and -802-5p, are predicted to interact with Wnt signaling genes, such as *Fzd8*, *Fzd10*, *Wnt7b*, and *Wnt10b*, that also have a reported role in fibrosis [54].

In mice, circulating levels of these miRNA two or five days post-exposure were associated with statistically significant differences in survival, depending on the strain and time point (Fig 7). Collectively, these results suggest (1) that changes in systemic miRNA expression within the first week following WTLI exposure can be used to predict negative health outcomes that do not begin to manifest until months later; (2) that the initial transient differences in the circulating miRNA profile following radiation injury have functional consequences for the animal; and, (3) that analysis of the differentially expressed miRNA may enable prediction of the onset of disease.

## Conclusion

To our knowledge, this is the first study reporting changes in the circulating and tissue-specific miRNA profile after partial body radiation. Characteristic and meaningful fluctuations in the circulating miRNA profile such as these during the first few days post-irradiation suggests that it may be possible to identify markers that predict increased risk of late organ injury during triage after a radiological incident. The relative ease in extracting and processing circulating miRNAs, their stability and tissue specificity together make circulating miRNA a promising class of biomarkers. Moreover, many of the miRNA that were differentially expressed in this study are already implicated in molecular pathways consistent with late organ injury. Identifying the underlying mechanisms associated with radiation-induced injury could do both, drive the successful search for tissue-damage specific biomarkers and reveal potential targets for intervention.

## Supporting information

S1 Fig Experimental set up for mouse whole thoracic lung irradiations. A Gulmay Medical RS320 Irradiation System X-ray unit operated at 300 kV and 10 mA giving a dose rate of 1.568 Gy/min (Gulmay Medical Ltd., Camberly, Surrey, UK) with a permanent 4 mm Be filter was used. The beam is filtered using 1.5 mm Cu and 3 mm Al giving a HVL of 3 mm Cu. (A) X-Ray unit holding the jig and lead lid; (B) empty jig; (C) jig and mice with (D) lead shielding exposing only the thorax (2.75 cm).
(PPTX)

S1 File Differential expression data for plasma miRNA. Tabulated results of differential expression analysis of plasma miRNA comparing irradiated animals to sham irradiated controls at the indicated times post-WTLI.
(CSV)

S2 File Differential expression data for tissue miRNA. Tabulated results of differential expression analysis of miRNA from the indicated tissue comparing irradiated animals to sham irradiated controls at the study endpoints (month 4 or month 6 post-WTLI for C3H or C57Bl/6 mice, respectively).
(CSV)

S1 Checklist (DOCX)
