# Supplementary material for: Identification of miRNA signatures associated with radiation-induced late lung injury in mice
Source: PLoS One. 2020 May 11;15(5):e0232411. doi: 10.1371/journal.pone.0232411 (PMC7213687; doi:10.1371/journal.pone.0232411)
Supplement: S1 Fig — A Gulmay Medical RS320 Irradiation System X-ray unit operated at 300 kV and 10 mA giving a dose rate of 1.568 Gy/min (Gulmay Medical Ltd., Camberly, Surrey, UK) with a permanent 4 mm Be filter was used. The beam is filtered using 1.5 mm Cu and 3 mm Al giving a HVL of 3 mm Cu. (A) X-Ray unit holding the jig and lead lid; (B) empty jig; (C) jig and mice with (D) lead shielding exposing only the thorax (2.75 cm). (PPTX) [file pone.0232411.s001.pptx]

## Slide 1
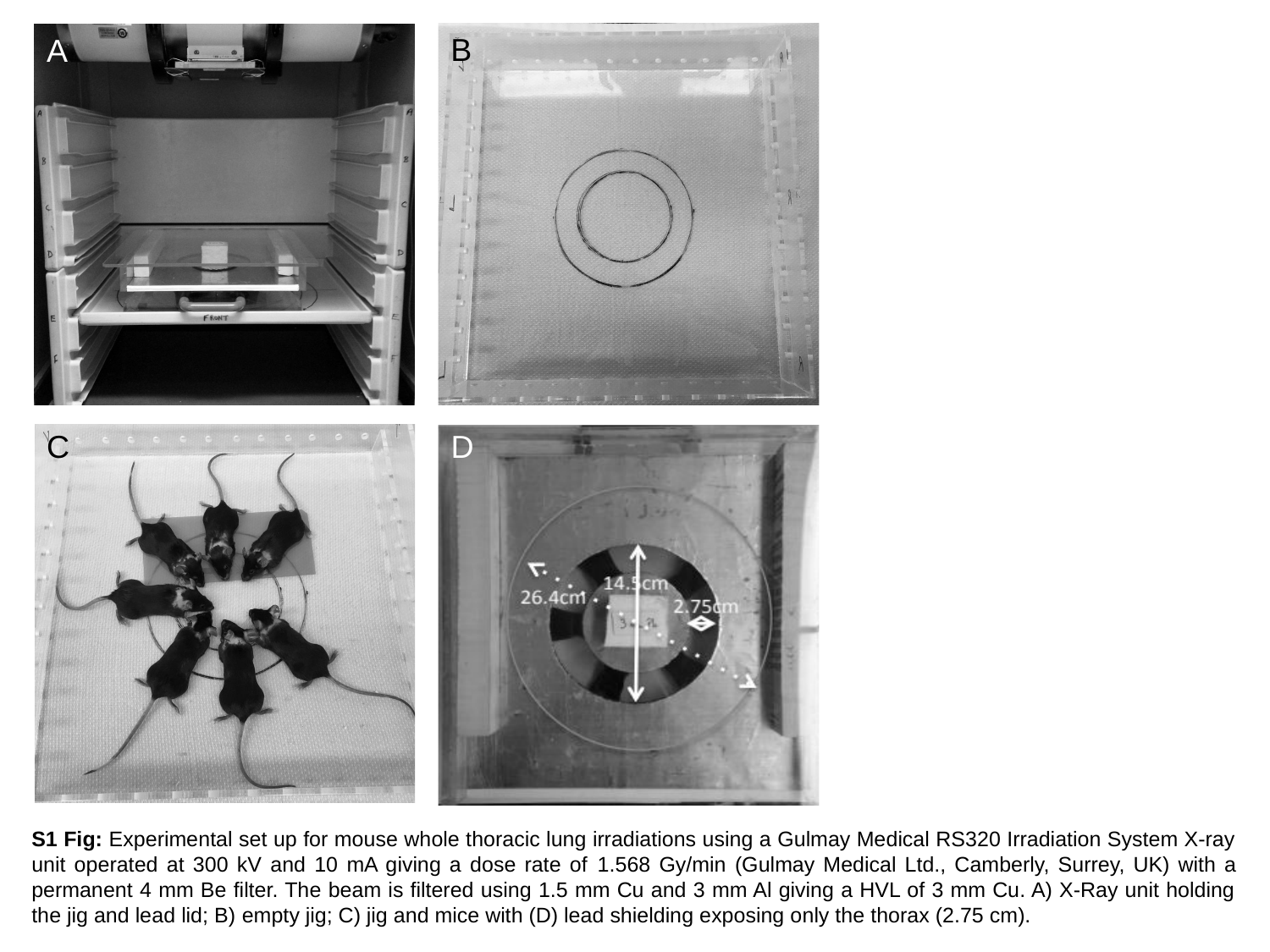

B
A
C
D
S1 Fig: Experimental set up for mouse whole thoracic lung irradiations using a Gulmay Medical RS320 Irradiation System X-ray unit operated at 300 kV and 10 mA giving a dose rate of 1.568 Gy/min (Gulmay Medical Ltd., Camberly, Surrey, UK) with a permanent 4 mm Be filter. The beam is filtered using 1.5 mm Cu and 3 mm Al giving a HVL of 3 mm Cu. A) X-Ray unit holding the jig and lead lid; B) empty jig; C) jig and mice with (D) lead shielding exposing only the thorax (2.75 cm).
